# The effect of PN-1, a Traditional Chinese Prescription, on the Learning and Memory in a Transgenic Mouse Model of Alzheimer's Disease

**DOI:** 10.1155/2013/518421

**Published:** 2013-02-17

**Authors:** Zhi-Gang Yao, Ling Zhang, Liang Liang, Yu Liu, Ya-Jun Yang, Lan Huang, Hua Zhu, Chun-Mei Ma, Chuan Qin

**Affiliations:** Comparative Medical Center, Institute of Laboratory Animal Science, Peking Union Medical College (PUMC), Chinese Academy of Medical Science (CAMS), Beijing 100021, China

## Abstract

Traditional Chinese Medicine (TCM) is a complete medical system that has been practiced for more than 3000 years. Prescription number 1 (PN-1) consists of several Chinese medicines and is designed according to TCM theories to treat patients with neuropsychiatric disorders. The evidence of clinical practice suggests the benefit effects of PN-1 on cognitive deficits of dementia patients. We try to prove and explain this by using contemporary methodology and transgenic animal models of Alzheimer's disease (AD). The behavioral studies were developed to evaluate the memory of transgenic animals after intragastric administration of PN-1 for 3 months. Amyloid beta-protein (A**β**) neuropathology was quantified using immunohistochemistry and ELISA. The western blotting was used to detect the levels of plasticity associated proteins. The safety of PN-1 on mice was also assessed through multiple parameters. Results showed that PN-1 could effectively relieve learning and memory impairment of transgenic animals. Possible mechanisms showed that PN-1 could significantly reduce plaque burden and A**β** levels and boost synaptic plasticity. Our observations showed that PN-1 could improve learning and memory ability through multiple mechanisms without detectable side effects on mice. We propose that PN-1 is a promising alternative treatment for AD in the future.

## 1. Introduction

Cognitive impairment or dementia in the elderly is associated with many disorders, such as Alzheimer's disease (AD) and vascular dementia (VaD) [1]. According to the traditional Chinese medicine (TCM), there is no distinction between AD and VaD. The term “senile dementia” refers to a clinical syndrome characterized by the progressive decline of memory and some related cognitive functions in the elderly [2]. In the ancient Chinese medicine literature, senile dementia falls into the category of “dementia,” or “idiocysyndrome” resulting from deficiency of kidney essence and marrow, obstruction by phlegm and blood stasis, and so forth [3, 4]. According to these traditional viewpoints, prescriptions composed of a complex variety of many different herbs, minerals, or animal parts are used to treat dementia clinically, such as Six Flavors Rehmannia Pills (*Liu Wei Di Hang Wan*), Nourish the Heart Decoction (*Yang Xin Tang*) and Gastrodia and Uncaria Drink (*Tian Ma Gou Teng Yin*) [5]. Prescription number 1 (PN-1) is a compound Chinese medicine designed entirely based on TCM theories. From a western medical view, this prescription has a wide range of pharmacological activities in clinical application to treat cerebellar atrophy, dystaxia, and cerebral palsy with minor side effects. In addition, the clinical effectiveness on poor memory of the elderly suggests a potential role of PN-1 in dementia therapeutics. Indeed, the efficacy of PN-1 in senile dementia was demonstrated by a nonblinded, open-label design performed by the Beijing Xuanwu Hospital in China (unpublished data).

As the most common type of dementia in western medicine, AD is a neurodegenerative disorder characterized clinically by progressive memory loss and neuropathologically by extracellular amyloid plaques [6]. The plaques are primarily composed of aggregated *β*-amyloid (A*β*) peptides derived from the *β*-amyloid precursor protein (APP) [7, 8]. Recent studies indicate that the increased A*β* production in the hippocampus and cortex leads to synaptic impairment, neuronal loss, and memory deficits [9–11]. Indeed, synaptic dysfunction was found in the association cortices and hippocampus of AD brain [12]. These findings suggest the excess production of A*β* peptides in the brain is a central event in AD pathology [13, 14]. In addition, synapse is the basis of learning and memory [15]. Hence, reactivating synaptic function is the key to improve learning and memory. In this study, we detected the effects of PN-1 on memory-associated behavior tests in transgenic mouse models of AD. Moreover, A*β* burden and synaptic function were investigated to understand the benefits of PN-1 to learning and memory deficits.

## 2. Material and Methods

### 2.1. Transgenic Mice

The APPswe/PS1dE9 (APP/PS1) transgenic mouse model of AD overexpresses the Swedish (K594M/N595L) mutation of APP together with presenilin 1 (PS1) deleted in exon 9 in a C57BL/6J genetic background. These mice show learning and memory deficits and increased senile plaques in behavioral and neuropathological analyses [16, 17]. The use of animals was in compliance with the National Institutes of Health Guide for Care and Use of Laboratory Animals. The study protocol was approved by the Institutional Animal Care and Use Committee of the Institute of Laboratory Animal Science (permit number: ILAS-PL-2010-004). The study and all procedures were conducted in accordance with institutional guidelines. All efforts were made to minimize suffering.

### 2.2. Preparation for PN-1

 PN-1 is composed of more than 20 kinds of Chinese medicines, in which the main herbs used are Radix Astragali, Radix Codonopsis, Rhizoma Atractylodis Macrocephalae, and Cistanches Herba in a dry weight ratio of 8 : 2 : 3 : 2. All components were authenticated macroscopically and microscopically according to pharmacopoeias [18]. In details, macroscopic examinations included measurements of appearance, size, shape, color, texture, odor, taste, fracture, and other characteristics of a herb. Microscopic examinations determined characteristic elements of each herb in both tissue and powder forms. In powder analysis, each component was pulverized to 200 mesh in size, mounted on a microscope slide, cleared with chloral hydrate, lactochloral and/or sodium hypochlorite, and then examined for the presence, size, shape, and numbers of characteristic elements and inclusions such as vessels, calcium crystals, crystalline fibers, stone cells, and starch grains. The examination protocols followed the World Health Organization (WHO) Quality Control Methods for Medicinal Plant Materials [19], the Pharmacopoeia of the People's Republic of China (CP) [18]. A mixture of powdered materials (500 g) were extracted three times with total 20 L of distilled water in a hot-water bath for 3 h, and, after filtration, the filtrates were mixed together and adjusted to a final concentration of 0.1 mg/mL (equivalent to dry weight of raw materials). 

### 2.3. Experimental Procedures

 Concentrated PN-1 extract was dissolved or suspended in distilled water. Transgenic APP/PS1 mice at 5 months of age were randomized into the vehicle-treated group (vehicle) and three experimental groups, including PN-1 0.6 mg/kg (low dose), 1.2 mg/kg (middle dose), and 2.4 mg/kg (high dose) once a day. Mice in the vehicle-treated group were orally given distilled water. The doses of the PN-1 used in this study were converted into raw material weights and given orally to mice for 4 months. We also introduced a positive control group, which was given Aricept (generic name donepezil, 2 mg/kg once a day) orally. Aricept is approved by FDA for the clinical treatment of AD and is usually used as a positive control in experiments [20, 21]. In addition, the age-matched C57BL/6 wild-type (WT) mice were required as control. Twenty animals (10 males and 10 females) were used for each group. Body weight, food and water intakes were measured every two weeks until behavior tests. At 8 months of age, the novel object recognition (NOR) task was performed to detect recognition memory of mice. One week after NOR task, spatial learning and memory of animals were assessed by the Morris water maze (MWM) test. Thereafter, all mice were sacrificed by decapitation under intraperitoneal injection anesthesia using sodium pentobarbital (45 mg/kg).

### 2.4. Novel Object Recognition Task

The NOR task consisted of a habituation phase, a training phase, and a testing phase. During habituation, each mouse was habituated to the open-field apparatus (30 cm wide, 45 cm long, and 20 cm high) made of polyvinyl chloride plastic for 5 min daily on 2 consecutive days in the absence of objects. No data were collected during habituation. In the training trial, mice were placed in the experimental apparatus and allowed to freely explore the arena in the presence of two identical objects (blue wooden cubes of side 3 cm) for 5 min daily on 3 consecutive days. The test phase was performed 24 h later. Each mouse was placed in the arena with an object they explored during the training phase (familiar object) and a new (novel) object (a yellow wooden cylinder of diameter 3 cm and height 3 cm). The open-field arena and the objects were cleaned thoroughly between trials to ensure the absence of olfactory cues. A mouse was scored as exploring an object when its head was oriented toward the object within a distance of 1 cm or when the nose was touching the object. Sitting on or going around the objects was not considered exploratory behavior. The exploration time for the familiar (*T*_*F*_) or the new object (*T*_*N*_) during the test phase was videotaped and analyzed using the Noldus Ethovision XT software (Noldus Information Technology, Wageningen, The Netherlands). Memory was defined by the discrimination index (DI) for the novel object as the following formula: DI = (*T*_*N*_ − *T*_*F*_)/(*T*_*N*_ + *T*_*F*_) × 100%  [22, 23].

### 2.5. Morris Water Maze Test

 The Morris water maze (MWM) test was performed in a circular pool (100 cm in diameter) filled with water at a temperature of 22 ± 1°C. The water was colored opaque with powdered nonfat milk. An overhead video camera coupled to a computer and tracking software (Ethovision system, Noldus Information Technology, Wageningen, The Netherlands) was used to track movements. The tank was placed in a dimly light, sound proof test room with various visual cues. The pool was divided into four quadrants of the equal area. A white platform (6 cm in diameter and 29 cm high) was centered in one of the pool quadrants. One day prior to hidden platform test, the mouse was allowed to swim for 60 sec in the pool with the visible platform projecting 1 cm above the water surface. The mouse was then given two trial sessions each day for five consecutive days, during which the platform was left in the same position and submerged 0.5 cm below the water surface. The time taken to reach the platform (escape latency) was measured, and the average of two trials was determined. 24 h after the last trial of the hidden platform test, the mouse was subjected to a probe trial in which the platform was removed from the pool, allowing the mouse to swim for 60 seconds in search of it. The frequency of target platform crossings was recorded. After the swim, the mouse was kept dry in a plastic holding cage on an electric heater.

### 2.6. Assessment of Alzheimer Pathology

 Brains were fixed in formalin and embedded in paraffin. For each specimen, 30 serial sections of 5 *μ*m thickness were coronally sliced for two to three such series, spaced 50 *μ*m apart. Sections were deparaffinized in xylenes and rehydrated via an ethanol gradient. Antigen retrieval was performed using 88% formic acid treatment for 5 min and incubated for 30 min with 0.3% H_2_O_2_ thereafter. Sections were blocked with 10% goat serum and incubated with primary antibody overnight at 4°C. The immunoreaction was visualized using horseradish peroxidase (HRP) labeled IgG as secondary antibody incubated at 37°C for 30 min and followed by diaminobenzidine (ZSGB, Beijing, China) staining. For the quantification of plaque levels, microscopic images of A*β* monoclonal antibody, 6E10 (1 : 1,000, Covance/Signet Laboratories, Dedham, MA)-stained cortex, and hippocampus were captured. The potential subregional variations of cortex and hippocampus were systematically analyzed. For thioflavin-S staining, the dewaxed sections were immersed in 1% thioflavin-S solution for 5 min and decolorized with 2 washes of 50% ethanol followed by washing and dehydration in increasing ethanol concentrations from 70% to 100%. The quantification of plaque number and size after immunostaining were analyzed using Aperio's ImageScope Viewer software (Aperio. Technologies). Thioflavin-S stained sections were observed by an Olympus BX40 microscope (Olympus, Tokyo, Japan), and the signal intensity was analyzed using ImageJ software (1.43u, NIH, USA). 

### 2.7. Quantitation of A*β* in Brain Extracts

The extraction of soluble and insoluble A*β* species (including A*β*40 and A*β*42) of the cortex and hippocampus homogenates was described in previous studies [24–26]. Briefly, The frozen mouse cortex and hippocampus were weighed and homogenized with ice-cold Trisbuffered saline (TBS) consisting of 20 mM Tris-HCl, 150 mM NaCl, and pH 7.4 to the frozen cortex at 4 : 1 (TBS volume/brain wet weight). The homogenate was centrifuged at 4°C for 30 min at 20,000 g. The supernatant containing soluble A*β* peptide fraction (called TBS extract) was aliquoted and then stored at −80°C, and the pellet containing insoluble A*β* was sonicated in an equal volume (v/v) of TBS plus 5 M guanidine HCl, pH 8.0, and incubated for 3-4 h at room temperature. The homogenate were centrifuged at 4°C for 30 min at 20,000 g. The supernatant was collected (called GuHCl extract) and regarded as the insoluble A*β* peptide fraction. Protein concentrations were estimated in both fractions using the NanoDrop 2000 spectrophotometer (Thermo Scientific, Wilmington, DE, USA). A*β*40 and A*β*42 levels were quantified by ELISA according to the manufacturer's recommendations (Invitrogen, CA, USA).

### 2.8. Western Blotting Analysis

 Brains tissue samples were homogenized using RIPA lysis buffer (50 mM Tris-HCl pH 7.4, 150 mM NaCl, 1% Triton X-100, 1% sodium deoxycholate, and 0.1% SDS) containing 1 mM PMSF. Total proteins from brain samples were measured using the NanoDrop 2000 spectrophotometer (Thermo Scientific, Wilmington, DE, USA). Equal amounts of protein were loaded on a 10% SDS-PAGE run at 90 V for 1.5 h at room temperature and transferred onto polyvinylidene fluoride (PVDF) membranes (Millipore, Billerica, MA, USA) and then probed with the primary antibodies overnight at 4°C. The primary antibodies and secondary antibodies were selected from the antibodies listed in Supplemental Table S1, available online at http://dx.doi.org/10.1155/2013/518421 including their dilution and commercial supplier. Then, membranes were incubated with peroxidase-labeled secondary antibody at room temperature for 1 h. Bands were visualized using enhanced chemiluminescent (ECL) substrate (Pierce, Rockford, IL USA) and quantitated by densitometry using NIH ImageJ software (1.43u, NIH, USA). Glyceraldehyde-3-phosphate dehydrogenase (GAPDH) was used as a loading control. Specific bands were quantified densitometrically, and the ratio between intensity of phospho-CaMKII*α*  and CaMKII*α*, CREB and phospho-CREB from the same homogenate were calculated.

### 2.9. Assessment of PN-1 Toxicity

Blood was collected after sacrifice by cardiopuncture. Serum was collected by clotting blood specimens for 10 min at room temperature followed by centrifugation at 2000 g for 10 min at 4°C. Serum was immediately subjected to total bilirubin (TB), alanine aminotransferase (ALT), aspartate aminotransferase (AST), creatinine (Cr), and blood urea nitrogen (BUN) analysis using commercial enzyme assays according to the manufacturer's instructions (Kang-Lan biotechnology Co. Ltd., Beijing). Tissues of liver, kidney, brain, and so forth, were collected after sacrifice. After fixation with 10% formaldehyde for 48 h, tissues were embedded in paraffin according to routine procedures. Five *μ*m thick sections were cut and stained with hematoxylin-eosin (H&E) for histopathological evaluation. Two expert pathologists at the Institute of Laboratory Animals blindly analyzed the tissue slices.

### 2.10. Statistical Analysis

All data were presented as means ± SEM. The group differences of escape latencies in the MWM test were analyzed using two-way ANOVA with repeated measures. Treatments were compared using a one-way ANOVA followed by Dunnett's multiple-comparison post hoc test using the GraphPad Prism. Significant differences were determined at *P* < 0.05.

## 3. Results

### 3.1. PN-1 Treatment Improves Recognition Memory Deficits in APP/PS1 Mice

**Figure 1:**
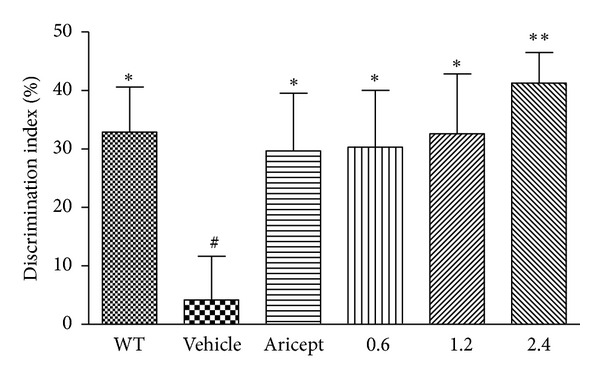
Effects of PN-1 treatment (mg/kg once a day) on the performance of the novel object recognition (NOR) task. Data represent means ± SEM (*n* = 17 mice/group). *P < 0.05, **P < 0.01 versus the vehicle-treated group; ^#^ *P* < 0.05 versus the wild-type group.

 As shown in Figure 1, the discrimination index (DI) was significantly reduced by 87% for vehicle-treated group compared to the wild-type (WT) group (*P* < 0.05). When compared with the vehicle-treated group, DI was significantly increased in Aricept- and PN-1-treated groups by approximately 6–8 folds (*P* < 0.05 for the Aricept, low- and middle-dose group; *P* < 0.01 for the high-dose group). 

### 3.2. PN-1 Treatment Improves Spatial Memory Deficits in APP/PS1 Mice

**Figure 2:**
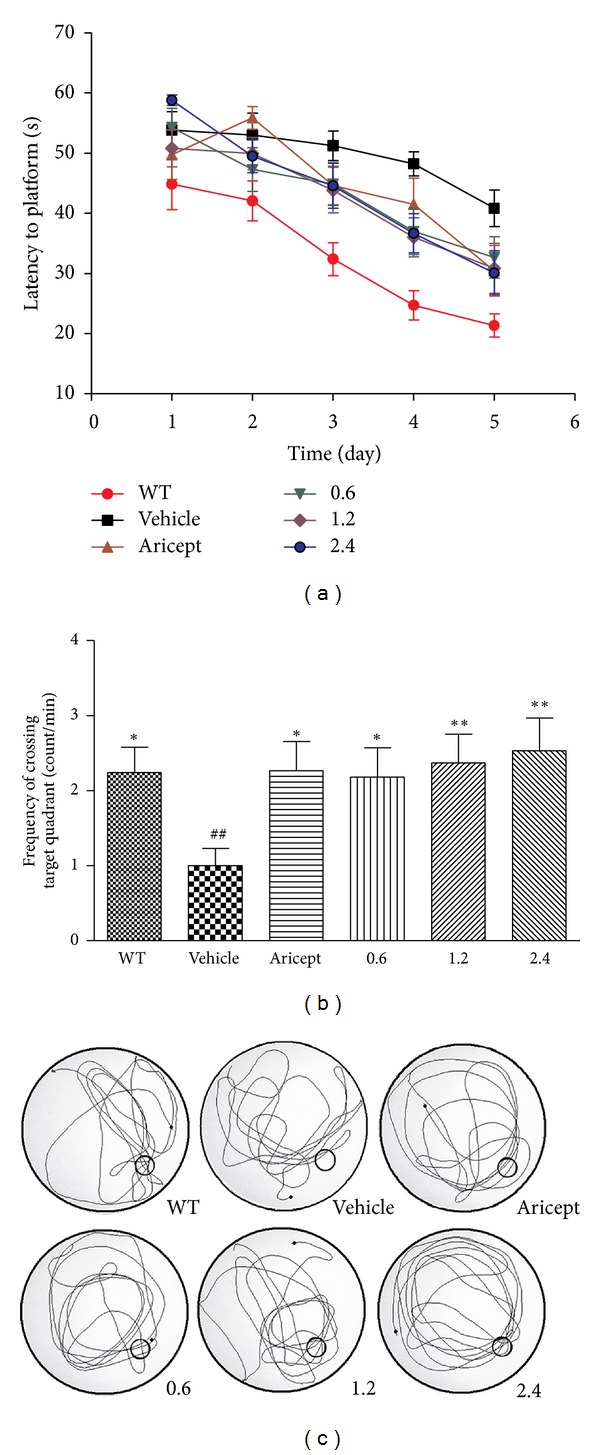
Effects of PN-1 treatment (mg/kg once a day) on the performance of the Morris water maze test. (a) The escape latency time in 5 days in the hidden platform test. Each point represents the mean latency of two trials per day for 5 consecutive days of testing. (b) The frequency of crossing within the platform quadrant detected in the probe trial. (c) An illustration of typical behavioral traces in the probe trial. Data represent means ± SEM (*n* = 17 mice/group). *P < 0.05, **P < 0.01 versus the vehicle-treated group; ^##^ *P* < 0.01 versus the wild-type group.

 In the hidden platform test (Figure 2(a)), the vehicle-treated group showed significantly increased escape latencies from day 2 compared to the wild-type group (*P* < 0.05 for day 2; *P* < 0.01 for day 3–5). When compared with the vehicle-treated group, escape latencies were significantly decreased in PN-1-treated groups from day 4 (*P* < 0.05 for day 4-5). At day 5, the Aricept-treated mice also showed significantly decreased escape latency (*P* < 0.05). In the probe trial (Figure 2(b)), vehicle-treated group showed significantly decreased frequency of crossing within the platform quadrant compared to wild-type group (*P* < 0.01). When compared with the vehicle-treated group, the frequency was increased in Aricept- and PN-1-treated groups (*P* < 0.05 for the Aricept and low-dose group;  *P* < 0.01  for the middle- and high-dose group). The typical behavioral traces from each group in the probe trial appear in Figure 2(c).

### 3.3. PN-1 Treatment Reduces Plaque Burden in the Brains of APP/PS1 Mice

**Figure 3:**
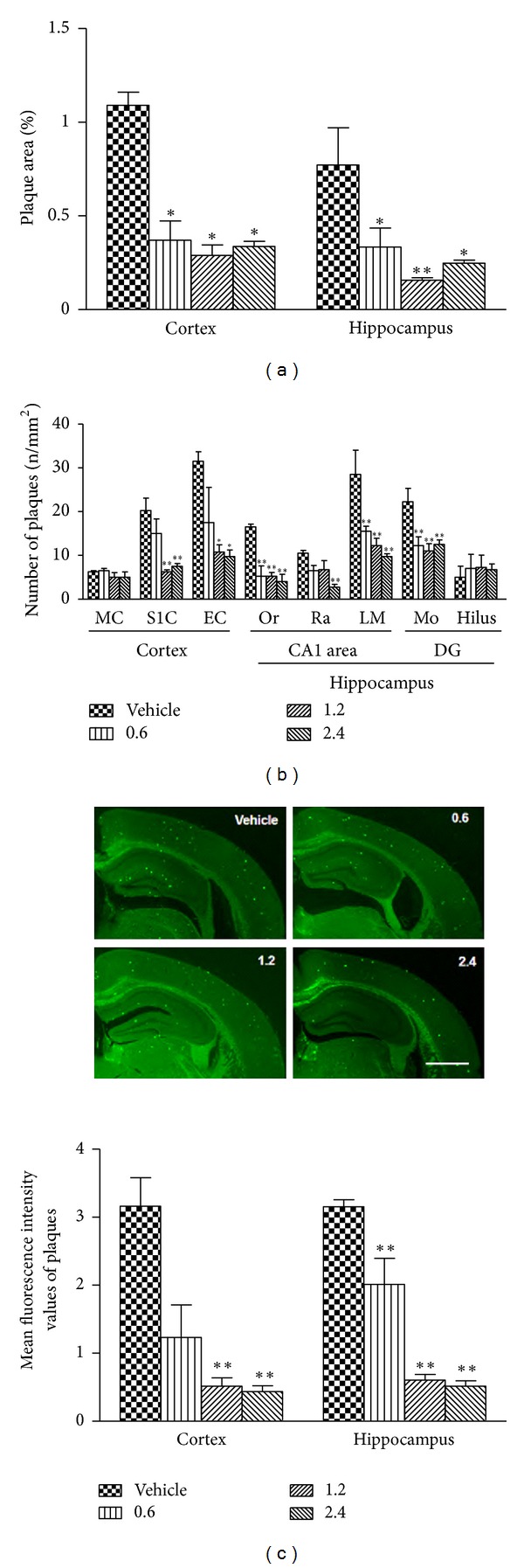
Effects of PN-1 treatment (mg/kg once a day) on plaque pathology. (a) The percentage area occupied by plaques in the cortex and hippocampus. (b) The average number of plaques per square millimeter in the cortical primary sensory cortex (S1C), entorhinal cortex (EC), and motor cortex (MC) and hippocampal stratum oriens (Or), stratum radiatum (Ra), stratum lacunosum-moleculare (LM), molecular layer (Mo), and dentate gyrus (DG) hilus. (c) The mean fluorescence intensity of thioflavin-S positive staining plaques in the cortex and hippocampus (scale bar, 1 cm). Data represent means ± SEM (*n* = 6 mice/group) from 2 and 3 independent experiments. *P < 0.05, **P < 0.01 versus the vehicle-treated group.

 Globally, results showed no A*β* deposition in wild-type brains (figure not shown). As shown in Figure 3(a), results showed significant reduction in the percentage of cortical and hippocampal area occupied by amyloid plaques in PN-1-treated groups compared to the vehicle-treated group (*P* < 0.05  for all PN-1-treated groups). In the cortex, the area of plaques was reduced by nearly 69% in PN-1-treated groups (*P* < 0.01  for all PN-1-treated groups). In hippocampus, PN-1 treatment also decreased the area of plaques by 57%–80% in PN-1-treated groups (*P* < 0.05  for the low- and high-dose group;  *P* < 0.01  for the middle-dose group). Moreover, the staining distribution pattern of plaques was distinguishable between groups (Figure 3(b)). PN-1 treatment showed significant reduction of plaque number in the primary sensory cortex (S1C,  *P* < 0.01  for the middle- and high-dose group) and entorhinal cortex (EC,  *P* < 0.05  for the middle- and high-dose group) by approximately 60%–70%, as compared with the vehicle-treated group. In the hippocampus, the plaque number in the stratum oriens (Or,  *P* < 0.01  for all PN-1-treated groups), stratum radiatum (Ra,  *P* < 0.01  for the high-dose group), and stratum lacunosum-moleculare (LM,  *P* < 0.01  for all PN-1-treated groups) of CA1 area and the molecular layer (Mo,  *P* < 0.01  for all PN-1-treated groups) of dentate gyrus (DG) was also significantly decreased by nearly 70%, 70%, 60%, and 45%, respectively. However, there is no significant difference in the number of plaques in the motor cortex (MC) and hippocampal hilus (polymorphic layer of DG). In addition, PN-1 treatment showed the significant effectiveness on plaque density decline (by nearly 80%) by assessing the signal intensity of thioflavin-S positive plaques in the cortex (*P* < 0.01  for the middle- and high-dose group) and hippocampus (*P* < 0.01  for all PN-1-treated groups) as shown in Figure 3(c). 

### 3.4. PN-1 Treatment Decreases A*β* Levels in the Brains of APP/PS1 Mice

**Figure 4:**
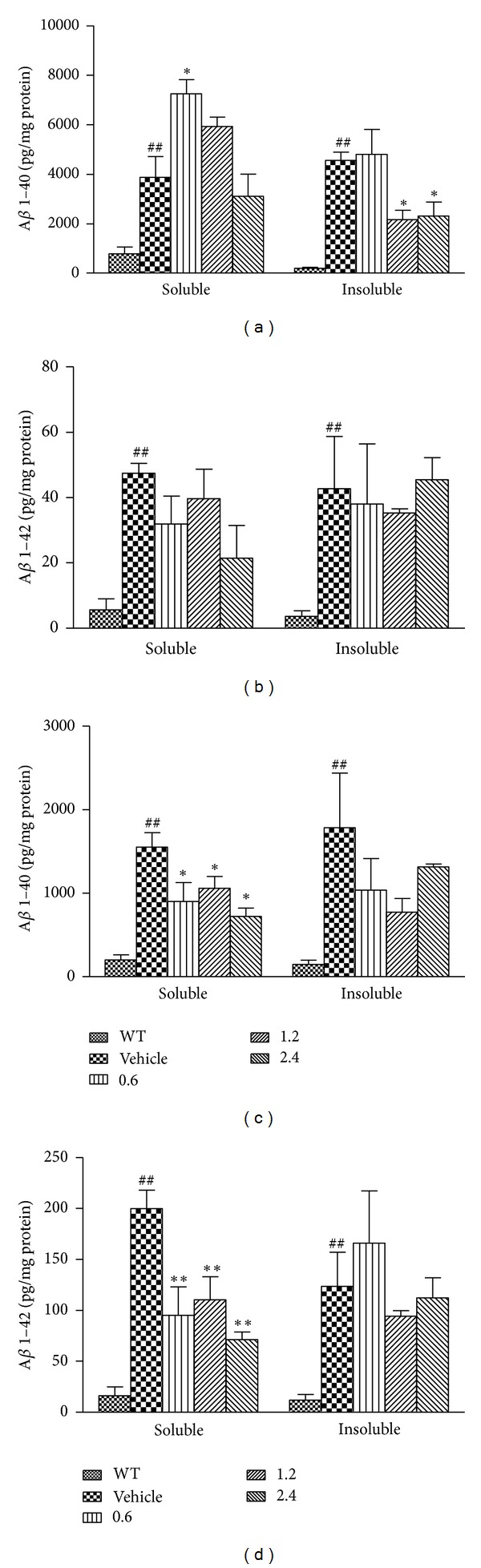
Effects of PN-1 treatment (mg/kg once a day) on A*β* levels. (a) The levels of cortical soluble and insoluble A*β*1–40. (b) The levels of cortical soluble and insoluble A*β*1–42. (c) The levels of hippocampal soluble and insoluble A*β*1–40. (d) The levels of hippocampal soluble and insoluble A*β*1–42. Data represent means ± SEM (*n* = 4  mice/group). *P < 0.05, **P < 0.01, versus the vehicle-treated group; ^##^ *P* < 0.01  versus the wild-type group.

 To extract and characterize A*β*1–40 and A*β*1–42 peptides present in mouse brains, we prepared soluble A*β* peptide fraction (TBS extract) and insoluble A*β* peptide fraction (GuHCl extract) by the sequential centrifugation of cortical and hippocampal homogenates. In the wild-type mouse brain, we detected much less soluble and insoluble A*β* peptides (Figure 4). As shown in Figure 4(a), the results showed the significant increase in soluble A*β*1–40 level in the low-dose group (*P* < 0.05) as well as the significant decrease (by nearly 50%) in insoluble A*β*1–40 level in the middle- and high-dose group (*P* < 0.05), as compared with the vehicle-treated group. For total cortical soluble and insoluble A*β*1–42 levels (Figure 4(b)), the results revealed no significant PN-1 treatment effects (*P* > 0.05  for all PN-1-treated groups). PN-1 treatment significantly lowered hippocampal total soluble A*β*1–40 (*P* < 0.05  for all PN-1-treated groups, Figure 4(c)) and A*β*1–42 (*P* < 0.01  for all PN-1-treated groups, Figure 4(d)) levels by approximately 40% and 50%, respectively. However, there was no significant effect of PN-1 on the levels of hippocampal insoluble A*β*1–40 (*P* > 0.05  for all PN-1-treated groups, Figure 4(c)) and A*β*1–42 (*P* > 0.05  for all PN-1-treated groups, Figure 4(d)). 

### 3.5. PN-1 Treatment Upregulates the Expressions of Plasticity-Related Proteins

**Figure 5:**
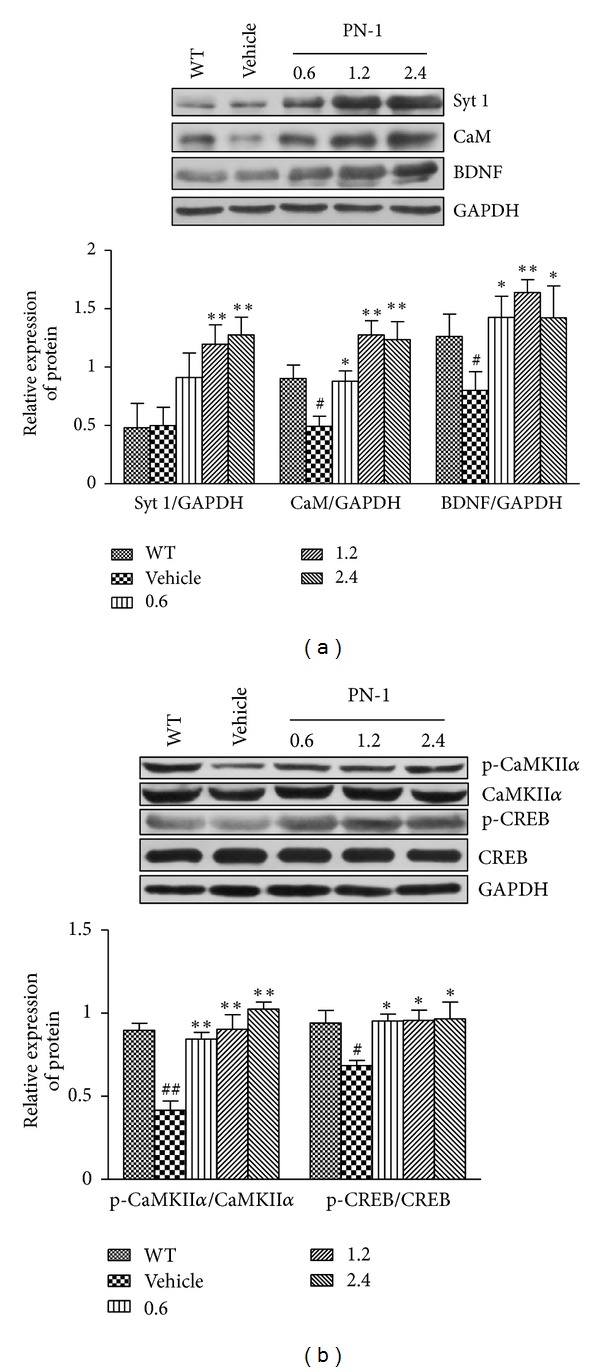
Effects of PN-1 treatment (mg/kg once a day) on the expressions of plasticity-related proteins. The top panels show a representative western blots, and the bottom panels show the quantitation of changes in protein levels by densitometry. (a) The levels of Syt 1, CaM, and BDNF expressions. (b) The phosphorylation status of CaMKII*α*  and CREB. Data represent means ± SEM (*n* = 6 mice/group) from 3 independent experiments. *P < 0.05, **P < 0.01  versus the vehicle-treated group. ^#^ *P* < 0.05, ^##^ *P* < 0.01  versus the wild-type group.

To further investigate the potential mechanisms underlying the beneficial effects of PN-1 on memory impairment of transgenic animals, presynaptic and postsynaptic proteins in the brain were analyzed using western blotting. As compared with the wild-type group, the levels of Syt 1, CaM, and BDNF expressions in the vehicle-treated group were decreased by 0%, 45%, and 37%, respectively (Figure 5(a),  *P* > 0.05  for Syt 1;  *P* < 0.05  for CaM and BDNF). The levels of Syt 1 in the middle- and high-dose of PN-1 treatment group were significantly upregulated by approximately 1.5-fold, as compared with the vehicle-treated group (*P* < 0.01). CaM levels were significantly increased by 78%, 159%, and 151% in the low-, middle-, and high-dose of PN-1-treated groups, respectively (*P* < 0.05  for the low-dose group,  *P* < 0.01  for the middle- and high-dose group). Compared to the vehicle-treated mice, BDNF levels nearly raised by 90% in all PN-1-treated groups (*P* < 0.05  for the low- and high-dose group,  *P* < 0.01  for the middle-dose group). 

Considering the crucial role of phosphorylated proteins in synaptic plasticity [27], we furtherly discussed the phosphorylation status of CaMKII*α*  and CREB (Figure 5(b)). The results showed no significant changes of CaMKII*α*  and CREB levels in each group after normalization using GAPDH (data not shown). As compared with the wild-type group, the ratios of p-CaMKII*α*/CaMKII*α*  and p-CREB/CREB in the vehicle-treated group were significantly declined by 46% and 27%, respectively (*P* < 0.01  for p-CaMKII*α*/CaMKII*α*;  *P* < 0.05  for p-CREB/CREB). In all PN-1-treated groups, the ratios of p-CaMKII*α*/CaMKII*α*  significantly raised by 79%, 91%, and 117%, respectively (*P* < 0.01 for all PN-1-treated groups). In addition, the ratios of p-CREB/CREB in PN-1-treated groups significantly raised by approximately 40% (*P* < 0.05  for all PN-1-treated groups).

### 3.6. PN-1 Treatment Shows No Detectable Side Effects on Mice

 During the long-term administration of PN-1, a battery of studies designed to detect possible side effects on animals. However, there was no detectable difference in body weight, food intake, or water intake between each group over the 3-month treatment period (Supplementary Figure  S1). It suggested that PN-1 did not influence the metabolic function of mice. Moreover, liver enzyme measurements and kidney function tests were also performed at the end of PN-1 administration. The results showed no significant effect on the liver or kidney function, as reflected by normal serum levels of total bilirubin (TB), alanine aminotransferase (ALT), aspartate aminotransferase (AST), creatinine (Cr), and blood urea nitrogen (BUN) (Supplementary Table 2). Microscopic examinations of the different tissues (brain, liver, kidney, lung, heart, spleen, etc.) showed no abnormal tissue architecture and cell morphology between vehicle- and PN-1-treated animals (Supplementary Figure S2).

## 4. Discussion

Although more than one hundred years had passed since AD was first described by Alois Alzheimer in 1906, the treatment is limited to the use of acetyl-cholinesterase inhibitors and N-methyl D-aspartic acid (NMDA) receptor antagonists [28]. In recent decades, many oriental medicine prescriptions have documented the efficacy and effectiveness in AD therapeutics, such as Yokukansan (Yi Gan San) [29–32] and Choto-san (Gou Teng San) [33–35]. They all contain a large number of components. These prescriptions exert potential benefits to cognitive functions in clinical trials and animal model studies and may unveil new strategy for dementia treatment as new beneficial candidates to widen therapeutic options for AD.

In this study, we first assessed the effect of PN-1 on the recognition memory for the NOR task, which is widely-used to assess nonspatial working, declarative memory task [36]. In our study, vehicle-treated mice could no longer discriminate a novel from a familiar object. However, Aricept- and PN-1-treated mice spent more time exploring novel objects versus familiar ones. The findings may suggest that PN-1 contributes to recognition memory consolidation. The MWM test has been used to assess hippocampal-dependent long-term spatial learning and memory [37, 38]. Vehicle-treated mice exhibited significantly impaired spatial learning and memory performance both in the hidden platform test and probe trial. However, this impairment was ameliorated after PN-1 treatment, suggesting that PN-1 may improve hippocampal-dependent long-term spatial learning and memory. Besides, NOR and MWM data provide additional evidence that PN-1 may have clinical beneficial effects on behavioral disturbances of Alzheimer-type dementia, such as getting lost behavior or misidentification [39, 40]. 

Since the accumulation of A*β* peptides in the brain is a central event of AD pathogenesis [13, 14] and strongly associates with cognitive decline [41], we next analyzed neuropathological changes after PN-1 treatment. The immunohistochemical results showed that PN-1 reduced plaque number and plaque size most effectively in the cortex and hippocampus of transgenic animals. Interestingly, the current data also demonstrated that PN-1 could reduce plaque number in subregions, such as cortical S1C and EC, hippocampal CA1 area, and DG molecular layer. In addition, we used thioflavin-S staining to detect dense-core plaques, which have more fibrillar A*β*42 with *β* sheet secondary structures associated with neuronal loss [42]. In our present study, the intensity of thioflavin-S positive staining decreased significantly following PN-1 treatment observed in the cortex and hippocampus. Furthermore, accumulating evidence has demonstrated that dense-core plaques associate with neuritic and inflammatory pathology in AD patients as well as in mouse models [43]. Therefore, our study also suggests the potential effects of PN-1 on neuroinflammation. Overall, neuropathological findings confirm that PN-1 can reduce amyloid plaque burden located in the brain of the transgenic mouse. We speculated that these changes are beneficial to learning and memory.

The fact that PN-1 affects the plaque burden suggests that it may influence A*β* metabolism in the brain. We observed suppressed soluble A*β* (including A*β*1–40 and A*β*1–42) levels in the hippocampus of PN-1-treated mouse. A recent study has shown that soluble A*β* is associated with AD [44]. Especially, the relative levels of A*β*42 are the key regulators of A*β* aggregation into amyloid plaques. Thus, A*β*42 has been implicated as the initiating molecule in the pathogenesis of AD [45, 46]. Additionally, A*β*40 associates with amyloid deposits in the cerebral vasculature (congophilic angiopathy, CAA) [47] and also causes age-dependent learning defects [48]. The decreased soluble A*β* level after PN-1 treatment may explain its ameliorating effects on hippocampus-dependent tasks of learning and memory tested by NOR and MWM. In contrast, neither hippocampal insoluble A*β*1–40 and A*β*1–42 levels nor cortical soluble A*β*1–40 and insoluble A*β*1–42 levels were changed by PN-1 treatments. We speculate the effects of PN-1 on A*β* levels are regional dependent, consistent with the immunohistochemical results in our study. Indeed, the inhomogeneous distribution of amyloid in the cerebral cortex was demonstrated in AD patients and animal models [49, 50]. Further studies are needed to investigate the possible mechanisms of PN-1 on A*β* metabolism, including A*β* production and clearance. In our study, endogenous A*β*40 and A*β*42 levels were also detected in the wild-type mouse brain, which coincides with previous reports [51].

Considering the toxic effects of soluble A*β* on synaptic functions [9–11], we next evaluated the actions of PN-1 on presynaptic and postsynaptic proteins. We first assessed the effect of PN-1 on CaMKII expression, which is a key mediator in regulating synaptic plasticity and long-memory formation [52]. The results showed there was no significant change in CaMKII*α*  expression among wild-type, vehicle-, and PN-1-treated mice. However, PN-1 treatment could strikingly increase phosphorylated CaMKII*α*  (Tyr286), which keeps CaMKII at an active state [53]. Then, the raised CaM, which is one of the upstream molecules in the CaMKII pathway and critical for CaMKII activation [54], was also detected in PN-1-treated mice. Here, both findings suggest that PN-1 may benefit the impaired long-term memory of transgenic mice through ameliorated CaM/CaMKII/CREB signaling pathway [55, 56]. This hypothesis was confirmed by the increased phosphorylation of CREB at Ser133, resulting in the activation of gene transcription and long-term memory formation [57, 58]. Evidence suggests that phosphorylation of CREB at Ser133 occurs in response to the BDNF expression [59]. The raised BDNF following PN-1 administration in our study suggests that BDNF is also involved in the effects of PN-1 on the improvement of learning and memory deficits in transgenic mice by regulating CaMKII/CREB system [60]. Interestingly, the raised synaptotagmin 1 (Syt 1) in PN-1-treated groups implies that PN-1 also has the potential impact on synaptic vesicles at presynaptic terminals, in which Syt 1 acts as a synaptic vesicle-associated protein that triggers Ca^2+^-sensitive, rapid neurotransmitter release [61, 62]. In this study, we also detected the expressions of N-Methyl-D-aspartate (NMDA) receptor 2B subunit (NR2B) and AMPA receptor GluR1 subunit. However, there was no significant difference of NR2B and GluR1 expressions among the six groups, suggesting that PN-1 may act on NMDA receptor and AMPA receptor through other mechanisms [63, 64]. Alternatively, the recruitment of NMDA receptor and AMPA receptor to postsynaptic membrane may also contribute to Ca^2+^ influx and subsequent activation of CaMKII to trigger synaptic plasticity observed in PN-1-treated mice abovementioned [65, 66], whereas the total amounts of NMDA receptor and AMPA receptor showed no significant change between each group. These data provide us with other research interests in the future. 

 We also examined the possible toxic side effects of PN-1 on the animals. Results showed no significant side effect of PN-1 on the metabolism, tissue structure, and function. In the main compositions of PN-1, Radix Astragali, as a traditional Chinese medicine, has been widely used for several thousand years with a lowtoxicity [67, 68]. Both Radix Codonopsis and Rhizoma Atractylodis Macrocephalae are usually used to protect neurons in brain ischemia [69, 70] and improve memory in dementia [71, 72] without significant side effect [72, 73]. Cistanches Herba could promote neuronal growth [74, 75] and enhance learning and memory of the mouse [74]. A new report, however, shows that Cistanches Herba induced cytotoxicity in the male reproductive systems of mice [76]. In our study, we found no evidence of toxicity to the male reproductive systems. According to the TCM theories, Chinese herbal prescriptions always contain numerous individual herbs, which work synergistically together to magnify their healing power and simultaneously counteract the potential side effects of other herbs involved [5]. Anyways, further studies are necessary to evaluate the long-term chronic toxicity of PN-1 in the future.

## 5. Conclusions

In summary, although the detailed chemical components and the exact targets of PN-1 are unknown now, the results of this study demonstrate that chronic treatment with PN-1 has a robust impact on memory deficits in transgenic mouse models of AD without detectable side effects. The potential mechanisms involved in the PN-1 actions include (1) reduction of amyloid burden in the cortex and hippocampus, (2) inhibition of hippocampal A*β* levels, and (3) reactivation of synaptic function-associated signal pathway. We think the present study has given us more insight into the actions of PN-1 in future research interests. Moreover, the development of such multicomponent herbal medicines targeting multiple sites could be useful for future drug discovery. Also, mechanism studies and identification of active compounds could lead to new discoveries in biological and biomedical sciences. These studies are underway.

## Supplementary Material

Supplementary Figure S1: Effects of PN-1 treatment (mg/kg once a day) on body weight (A), food intake (B) and water intake (C) in each group detected every two weeks during the 3-month treatment period. Data represent means ± SEM (n = 6 mice/group).
Supplementary Figure S2: Effects of PN-1 on morphology and architecture of liver, kidney and brain (including the cortex and hippocampus) by hematoxylin-eosin staining. The mice from the high-dose (2.4 mg/kg) of PN-1 treatment group showed essentially normal liver architecture with hepatocytes (H) radiating from the central vein (V), sinusoids (S). Sections of the kidney from the high-dose of PN-1 treatment group showed the normal architecture of tubules (T) and glomeruli (G). They also presented the typical layered appearance of the cerebral cortex (C) and hippocampal granular layer (GL) of CA1 area as well as neurons (N). n = 6 mice/group, Bar = 100 *µ*m.
Supplementary Table S1: List of antibodies used in the study.
Supplementary Table S2: Serum biochemical parameters to evaluate liver and kidney.
