# Supplementary material for: The effect of PN-1, a Traditional Chinese Prescription, on the Learning and Memory in a Transgenic Mouse Model of Alzheimer's Disease
Source: Evid Based Complement Alternat Med. 2013 Feb 17;2013:518421. doi: 10.1155/2013/518421 (PMC3588396; doi:10.1155/2013/518421)
Supplement: Supplementary file 1 — Supplementary Figure S1: Effects of PN-1 treatment (mg/kg once a day) on body weight (A), food intake (B) and water intake (C) in each group detected every two weeks during the 3-month treatment period. Data represent means ± SEM (n = 6 mice/group). Supplementary Figure S2: Effects of PN-1 on morphology and architecture of liver, kidney and brain (including the cortex and hippocampus) by hematoxylin-eosin staining. The mice from the high-dose (2.4 mg/kg) of PN-1 treatment group showed essentially normal liver architecture with hepatocytes (H) radiating from the central vein (V), sinusoids (S). Sections of the kidney from the high-dose of PN-1 treatment group showed the normal architecture of tubules (T) and glomeruli (G). They also presented the typical layered appearance of the cerebral cortex (C) and hippocampal granular layer (GL) of CA1 area as well as neurons (N). n = 6 mice/group, Bar = 100 µm. Supplementary Table S1: List of antibodies used in the study. Supplementary Table S2: Serum biochemical parameters to evaluate liver and kidney. [file 518421.f1.pdf]

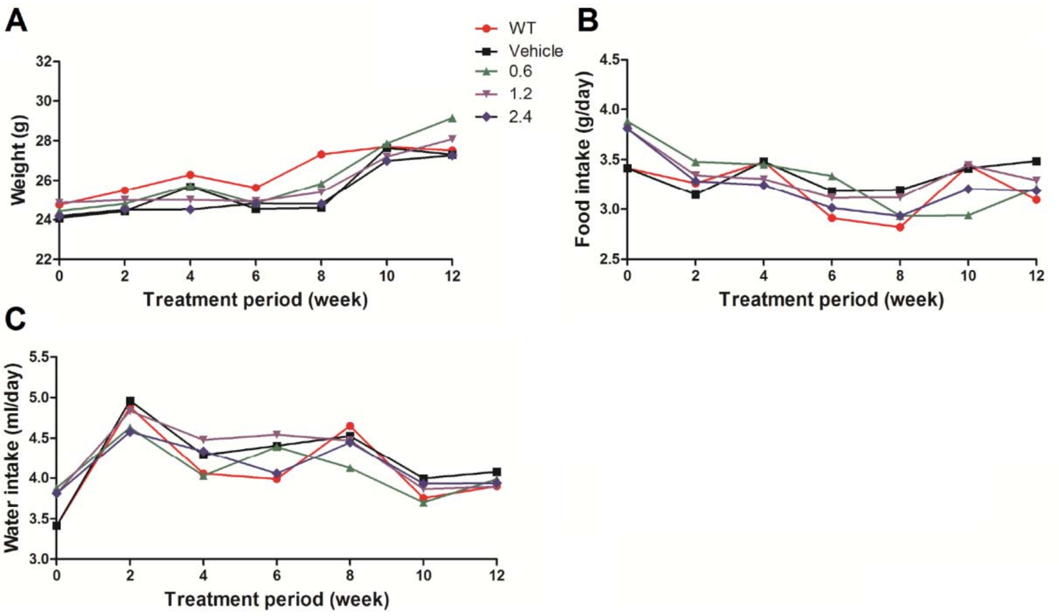

Supplementary FIGURE S1. Effects of PN-1 treatment (mg/kg once a day) on body weight (A), food intake (B) and water intake (C) in each group detected every two weeks during the 3-month treatment period. Data represent means ± SEM (n = 6 mice/group).
