# Supplementary material for: The effect of PN-1, a Traditional Chinese Prescription, on the Learning and Memory in a Transgenic Mouse Model of Alzheimer's Disease
Source: Evid Based Complement Alternat Med. 2013 Feb 17;2013:518421. doi: 10.1155/2013/518421 (PMC3588396; doi:10.1155/2013/518421)
Supplement: Supplementary file 2 [file 518421.f2.pdf]

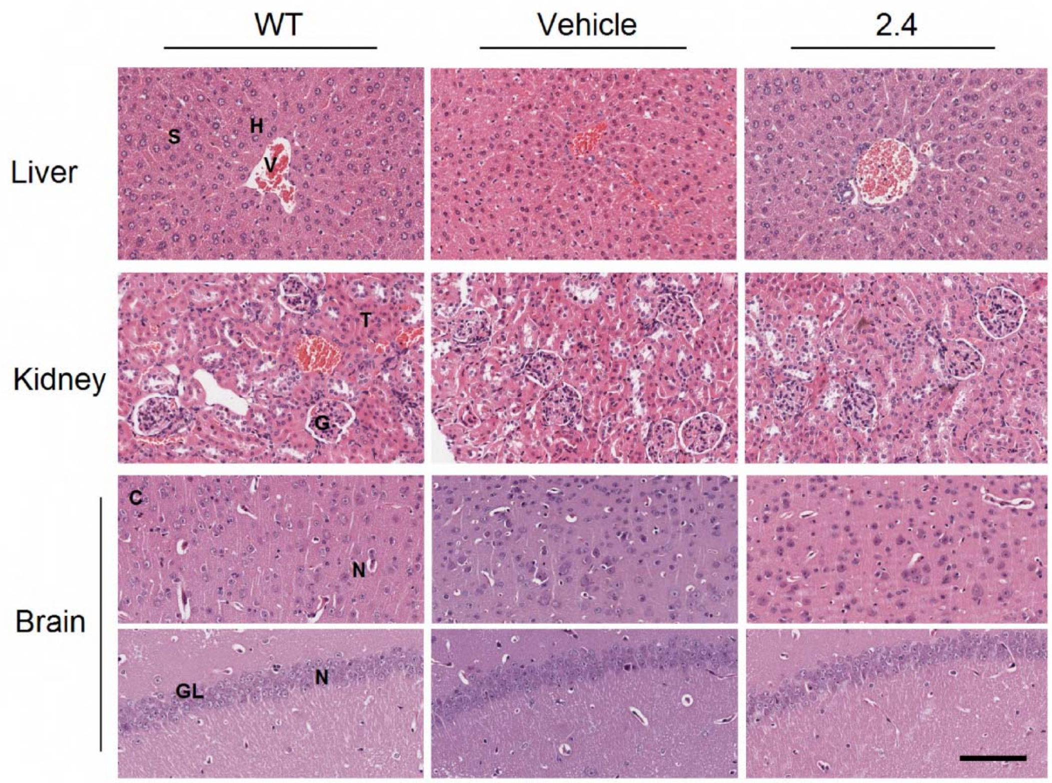

Supplementary FIGURE S2. Effects of PN-1 on morphology and architecture of liver, kidney and brain (including the cortex and hippocampus) by hematoxylin-eosin staining. The mice from the high-dose (2.4 mg/kg) of PN-1 treatment group showed essentially normal liver architecture with hepatocytes (H) radiating from the central vein (V), sinusoids (S). Sections of the kidney from the high-dose of PN-1 treatment group showed the normal architecture of tubules (T) and glomeruli (G). They also presented the typical layered appearance of the cerebral cortex (C) and hippocampal granular layer (GL) of CA1 area as well as neurons (N). n = 6 mice/group, Bar = 100 μm.
