# Supplementary material for: The effect of PN-1, a Traditional Chinese Prescription, on the Learning and Memory in a Transgenic Mouse Model of Alzheimer's Disease
Source: Evid Based Complement Alternat Med. 2013 Feb 17;2013:518421. doi: 10.1155/2013/518421 (PMC3588396; doi:10.1155/2013/518421)
Supplement: Supplementary file 3 [file 518421.f3.pdf]

**Supplementary TABLE S1:** List of antibodies used in the study.

| Antigen | Type | Dilution | Source | Catalog Number |
|---|---|---|---|---|
| Syt 1 | Rb pAb | 1:200 | abcam | ab51164 |
| CaM | Rb mAb | 1:1, 000 | abcam | ab45689 |
| CaMKIIα | Rb mAb | 1:1, 000 | abcam | ab92332 |
| p-CaMKIIα (Thr286) | Rb pAb | 1:1, 000 | abcam | ab32678 |
| CREB | Rb mAb | 1:500 | CST | 9197 |
| p-CREB (Ser133) | Rb mAb | 1:200 | CST | 9198 |
| BDNF | Rb pAb | 1:100 | abcam | ab72439 |
| HRP- conjugated anti-GAPDH IgG | | 1:10, 000 | ZSGB | TA-08 |
| HRP-conjugated anti-rabbit IgG | | 1:1, 000 | ZSGB | ZF-2301 |
| HRP-conjugated anti-mouse IgG | | 1:1, 000 | ZSGB | ZF-2305 |
