# Supplementary material for: The effect of PN-1, a Traditional Chinese Prescription, on the Learning and Memory in a Transgenic Mouse Model of Alzheimer's Disease
Source: Evid Based Complement Alternat Med. 2013 Feb 17;2013:518421. doi: 10.1155/2013/518421 (PMC3588396; doi:10.1155/2013/518421)
Supplement: Supplementary file 4 [file 518421.f4.pdf]

Supplementary TABLE 2: Serum biochemical parameters to evaluate liver and kidney function.

	WT	Vehicle	Aricept	0.6	1.2	[illegible]
TB (μmol/L)	1.006 ± 0.30	0.834 ± 0.40	1.65 ± 0.26	1.104 ± 0.16	1.044 ± 0.30	0.97[illegible]
ALT (U/L)	49.38 ± 13.88	39.1 ± 7.99	41.92 ± 7.83	41.28 ± 4.12	34.24 ± 3.37	38.4[illegible]
AST (U/L)	146.08 ± 33.33	139.36 ± 13.18	129.28 ± 20.11	137.82 ± 21.90	129.54 ± 21.38	137.4[illegible]
Cr (μmol/L)	9.81 ± 1.26	10.43 ± 0.46	9.13 ± 1.25	9.96 ± 1.42	9.91 ± 0.92	9.1[illegible]
BUN (mmol/L)	10.14 ± 1.77	9.60 ± 3.00	9.41 ± 0.77	8.68 ± 1.95	7.13 ± 1.16	8.7[illegible]

TB, total bilirubin, ALT, alanine aminotransferase, AST, aspartate aminotransferase, Cr, Creatinine, BUN, blood urea nitrogen
